# Emerging Enteroviruses Causing Hand, Foot and Mouth Disease, China, 2010–2016

**DOI:** 10.3201/eid2410.171953

**Published:** 2018-10

**Authors:** Yu Li, Zhaorui Chang, Peng Wu, Qiaohong Liao, Fengfeng Liu, Yaming Zheng, Li Luo, Yonghong Zhou, Qi Chen, Shuanbao Yu, Chun Guo, Zhenhua Chen, Lu Long, Shanlu Zhao, Bingyi Yang, Hongjie Yu, Benjamin J. Cowling

**Affiliations:** Chinese Center for Disease Control and Prevention. Beijing, China (Y. Li, Z. Chang, Q. Liao, F. Liu, Y. Zheng, L. Luo, S. Yu, H. Yu);; The University of Hong Kong, Hong Kong, China (Y. Li, P. Wu, B. Yang, B.J. Cowling);; Fudan University, Shanghai, China (Y. Zhou, H. Yu);; Hubei Center for Disease Prevention and Control, Wuhan, China (Q. Chen);; Huazhong University of Science and Technology, Wuhan, China (C. Guo);; Chengdu Center for Disease Prevention and Control, Chengdu, China (Z. Chen);; Sichuan University, Chengdu, China (L. Long);; Hunan Center for Disease Prevention and Control, Changsha, China (S. Zhao)

**Keywords:** hand foot and mouth disease, enterovirus, surveillance, China, coxsackievirus, viruses

## Abstract

Coxsackievirus A6 emerged as one of the predominant causative agents of hand, foot and mouth disease epidemics in many provinces of China in 2013 and 2015. This virus strain accounted for 25.9% of mild and 15.2% of severe cases in 2013 and 25.8% of mild and 16.9% of severe cases in 2015.

Hand, foot and mouth disease (HFMD) is a common childhood infectious disease caused by enteroviruses (*1*). In China, HFMD cases must be reported to the Notifiable Infectious Diseases Reporting Information System. Apart from clinical and demographic information, case notifications also include etiologic results, if available, classified into 3 categories: enterovirus A71 (EV-A71), coxsackievirus (CV) A16, and other enteroviruses. However, not all cases have etiologic results, the Notifiable Infectious Diseases Reporting Information System (NIDRIS) does not indicate cases that tested negative for enteroviruses, and testing methods vary among hospitals (*2*). To capture more information on the etiologic spectrum of HFMD in China, a laboratory surveillance network has been established in provincial-level centers for disease control and prevention (CDCs). EV-A71 and CV-A16 were previously believed to be the main causative viruses for HFMD in Asia, but several studies have suggested that other enteroviruses appear to be increasing since 2008 (*3**–**9*). Nevertheless, these past studies in China could not provide an overview at the national level because of limitations in geographic locations or study settings; furthermore, none of them systematically examined proportions of specific enteroviruses testing positive among tested HFMD cases. We analyzed data from this laboratory network to examine causative pathogens of HFMD cases and epidemiologic differences associated with various pathogens.

## The Study

Since June 2009, clinical specimens must be collected from all severe HFMD cases, and the first 5 reported mild cases (case classification criteria in the Technical Appendix) in each county of China every month and are tested for enteroviruses at local CDCs using PCR (Technical Appendix) (*10*). Test results are characterized as negative for enterovirus or positive for EV-A71, CV-A16, or other enteroviruses. For specimens testing positive for other enteroviruses, further serotyping is not conducted as a routine practice, but some local CDCs with more laboratory capacity may select a subset of these specimens to test on the serotype at their own discretion, especially when the proportion of other enteroviruses detected was relatively high.

We collected individual laboratory data during January 2010–December 2016 from 23 provincial CDCs (Technical Appendix Figure 1). In these provinces, HFMD case notifications accounted for 88.4% of HFMD cases notified in China overall. We analyzed virus serotypes in combination with sex, age, and clinical severity of each case. The dataset includes 693,580 individual illness episodes in the 23 provinces; 7,632–59,507 (median 31,317) episodes per province were reported. Clinical samples were collected from each illness onset, including throat swabs (374,685; 54.0%), feces (153,947; 22.2%), rectal swabs (129,837; 18.7%), and other specimens (35,111; 5.1%) such as vesicular or cerebrospinal fluid.

**Figure 1:**
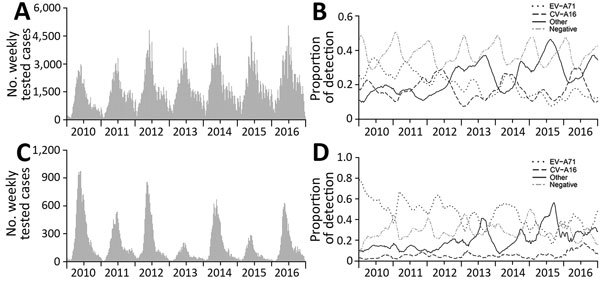
Weekly proportions of enteroviruses detection by serotype among hand, foot and mouth disease cases, January 2010–December 2016, China: A) number of tested mild cases; B) proportions of serotypes among mild cases C) number of tested severe cases; D) proportions of serotypes among severe cases.

Weekly proportions of positive enteroviruses (1 – enterovirus-negative specimens divided by all specimens collected for testing) were generally lower in mild cases (median 62.4%, range 42.0%–74.0%) than in severe cases (median 73.1%, range 27.3%–100%) and showed seasonal variations: peaks in April–May and low levels in December–January (Figure 1). The highest weekly proportion of EV-A71 detections among mild cases was 37.7% in 2010; a decreasing trend was observed thereafter. EV-A71 vaccine probably had little effect on the change in EV-A71 detections because it was not available until March 2016 and was not included in the routine vaccination program. In contrast, weekly proportions of detection of other enteroviruses generally increased with time, reaching a maximum of 48.4% in 2015. The proportion of cases positive for CV-A16 was relatively stable at ≈20% across the years, following a similar temporal trend to that of EV-A71. However, detections of different serotypes of enteroviruses generally demonstrated a similar temporal pattern among severe cases as among mild cases, except that the proportion of CV-A16 was relatively low, fluctuating at ≈5% across the period (Figure 1). Proportions of detection generally declined with age for other enteroviruses, whereas EV-A71 and CV-A16 showed an increasing trend with age, particularly in mild cases (Technical Appendix Figure 2).

**Figure 2:**
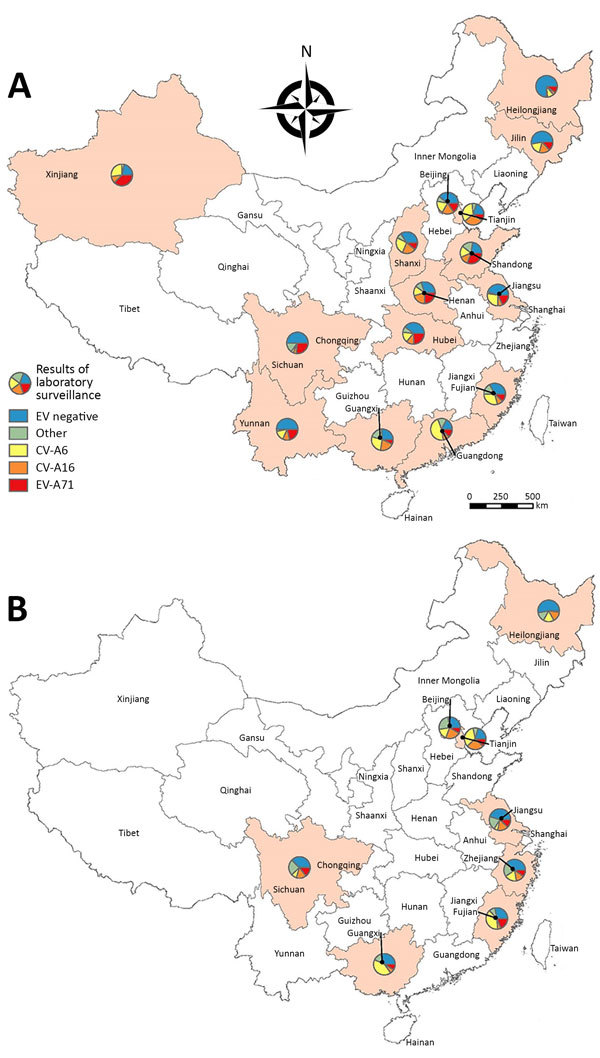
Estimated yearly detection proportions of CV-A6, EV-A71, CV-A16, and other enteroviruses among mild hand, foot and mouth disease cases by province in China: A) 2013; B) 2015. CV, coxsackievirus; EV, enterovirus.

EV-A71 and CV-A16 predominated in 2010–2012, 2014, and 2016, but other enteroviruses were predominant in 2013 and 2015. In those 2 years, further serotyping on other enteroviruses was widely conducted (Table). In 2013, a total of 3,260 (11.6% of 28,111 specimens positive for other enteroviruses) specimens collected from mild cases and 42 (4.3% of 983) specimens collected from severe cases underwent further serotyping; in 2015, a total of 2,474 (6.4% of 38,535) specimens collected from mild cases and 71 (3.9% of 1,822) specimens collected from severe cases underwent further serotyping. The serotyping results showed that, of mild cases infected with other enteroviruses, 80% in 2013 and 59% in 2015 were infected with CV-A6; for severe cases, 67% in 2013 and 44% in 2015 were infected with CV-A6. By multiplying the proportion of other enteroviruses by the proportion of CV-A6 among other enteroviruses, we estimated that CV-A6 accounted for 25.9% of mild cases and 15.2% of severe cases in 2013 and 25.8% of mild cases and 16.9% of severe cases in 2015. This result at the national level supports regional and subregional studies in China (*3**–**9*), suggesting that CV-A6 emerged as a main causative agent of HFMD in 2013 and 2015, but detections of CV-A6 were still low in some provinces of southwestern and northeastern China (Figure 2; Technical Appendix Figure 3). D3 is the predominant subgenotype for CV-A6 (*11*). During the same period, CV-A10 accounted for 4.2%–9.5% of other enteroviruses. Serotypes other than CV-A6 and CV-A10, including CV-A2, CV-A5, CV-A4, CV-B4, echovirus 6 and -25, and others, accounted for a small proportion (0.03%–2.4%) of specimens tested for further serotyping. These rare serotypes could possibly become prevalent in the future because of accumulative immunity to the prevalent enteroviruses, the potential replacement effect induced by vaccination programs against predominant enteroviruses, or both. One limitation of serotyping results of other enteroviruses is that they tend to reflect those of areas with more intensive HFMD transmission, because local CDCs generally select areas where the most cases are detected for further serotyping.

**Table:** Serotypes of non–EV-A71 and non–CV-A16 enteroviruses among HFMD cases, by clinical severity, 2013 and 2015, China*

Test result	2013, no. (%)			2015, no. (%)	
Mild, n = 87,226	Severe, n = 3,837	Mild, n = 99,461	Severe, n = 4,712
EV negative	32,801 (37.60)	1,100 (28.67)		35,167 (35.36)	1,273 (27.02)
EV-A71	15,503 (17.77)	1,557 (40.58)		11,800 (11.86)	1,352 (28.69)
CV-A16	10,811 (12.39)	197 (5.13)		13,959 (14.03)	265 (5.62)
Other enteroviruses	28,111 (32.23)	983 (25.62)		38,535 (38.74)	1,822 (38.67)
Further serotyping of other enteroviruses					
Total	Mild, n = 3,260†	Severe, n = 42‡		Mild, n = 2,474§	Severe, n = 71¶
CV-A6	2,620 (80.37)	28 (66.67)		1,471 (59.46)	31 (43.70)
CV-A10	176 (5.40)	4 (9.52)		104 (4.20)	#
CV-A2	22 (0.67)	1 (2.38)		5 (0.20)	#
CV-A5	9 (0.28)	1 (2.38)		2 (0.08)	#
CV-A4	2 (0.06)	#		9 (0.36)	#
ECV-6	9 (0.28)	#		#	#
CV-B4	13 (0.4)	#		#	#
ECV-25	8 (0.25)	#		#	#
CV-A12	5 (0.15)	#		#	#
CV-B5	5 (0.15)	#		#	#
CV-B2	4 (0.12)	#		1 (0.04)	#
ECV-7	4 (0.12)	#		#	#
ECV-9	3 (0.09)	#		#	#
CV-A8	1 (0.03)	#		3 (0.12)	#
CV-A14	2 (0.06)	#		#	#
CV-A21	2 (0.06)	#		#	#
ECV-12	2 (0.06)	#		#	#
CV-B1	2 (0.06)	#		#	#
ECV-30	1 (0.03)	#		1 (0.04)	#
Other	6 (0.18)**	#		#	#
Untyped	364 (11.37)	8 (19.05)		878 (35.49)	40 (56.34)

*CV, coxsackievirus; ECV, echovirus; EV, enterovirus. †Results are based on data from Beijing, Fujian, Guangdong, Guangxi, Heilongjiang, Henan, Hubei, Jiangsu, Jilin, Shanxi, Sichuan, Shandong, Tianjin, Xinjiang, and Yunnan provinces.  ‡Results are based on data from Fujian, Guangdong, Guangxi, Henan, and Shanxi provinces.  §Results are based on data from Beijing, Fujian, Guangxi, Heilongjiang, Jiangsu, Sichuan, Tianjin, and Zhejiang provinces.  ¶Results are based on data from Guangdong and Jiangsu provinces.  #The serotypes were not detected, but might be included in the untyped specimens because of laboratory capacity restrictions. **Other serotypes include CV-A20, CV-A24, ECV-1, ECV-96, Polio1, and Polio2, with 1 of each serotype detected.

We found that detection proportions of other enteroviruses were generally negatively associated with that of EV-A71 in mild and severe cases (Pearson correlation coefficient −0.73 for mild cases and −0.70 for severe cases) and CV-A16 in mild cases (Pearson correlation coefficient −0.52) (Figure 1). This result might indicate competitive interactions between other enteroviruses and EV-A71 or CV-A16, which should be considered when evaluating the effect of introducing a new enterovirus vaccine, especially when the proportion of other enteroviruses is increasing. The epidemiologic modeling study suggested that cross-protection between EV-A71 and CV-A16 exists for nearly 7 weeks, on average, in the context of natural infections (*12*). However, vaccine trials reported that monovalent EV-A71 vaccine failed to protect against CV-A16–associated HFMD (*13*). Similarly, whether cross protection exists between EV-A71 and other enteroviruses, such as CV-A6 and CV-A10, remains poorly understood to date, although limited studies have been more indicative of a lack of cross protection between EV-A71 and coxsackieviruses including CV-A6 (*14**,**15*).

## Conclusions

Data from national laboratory network surveillance of HFMD in China show that detection of enteroviruses other than EV-71 and CV-A16 has been increasing in both mild and severe cases and that CV-A6 has been emerging as another predominant serotype recently, but not in every province. Serotyping of individual enteroviruses apart from currently tested EV-71 and CV-A16 is suggested for routine virologic surveillance. Further studies may be needed to investigate potential cross immunity between EV-A71 and other enteroviruses such as CV-A6, CV-A10, and others.

Technical Appendix Criteria for classification of mild and severe cases of hand, foot and mouth disease and introduction to testing methods, China, 2010–2016.
