# Supplementary material for: Emerging Enteroviruses Causing Hand, Foot and Mouth Disease, China, 2010–2016
Source: Emerg Infect Dis. 2018 Oct;24(10):1902–6. doi: 10.3201/eid2410.171953 (PMC6154135; doi:10.3201/eid2410.171953)
Supplement: Technical Appendix — Criteria for classification of mild and severe cases of hand, foot and mouth disease and introduction to testing methods, China, 2010–2016. [file 17-1953-Techapp-s1.pdf]

Article DOI: https://doi.org/10.3201/eid2410.171953

Emerging Enteroviruses Causing Hand, Foot and Mouth Disease, China, 2010–2016

Technical Appendix

Criteria for Classification of Mild and Severe HFMD Cases

Consistent with a prior study (*1*), severe hand, foot and mouth disease (HFMD) cases are defined as HFMD cases developing neurologic complications (including aseptic meningitis, encephalitis, encephalomyelitis, acute flaccid paralysis, autonomic nervous system dysregulation, or a combination) and/or cardiopulmonary involvement such as pulmonary edema, pulmonary hemorrhage, or cardiorespiratory failure. HFMD cases without these complications are classified as mild cases.

Surveillance Network, Specimen Collection, and Testing Methods

Details on laboratory surveillance network, sample collection, and molecular methods have been introduced in a prior study (*1*), with specific details for this dispatch as follows:

Laboratory Surveillance Network

Since June 2009, each county or district center for disease control and prevention (CDC) must collect samples from at least the first 5 consecutive mild HFMD cases each month and from all severe cases. As of the time of this writing, 23 of 31 provinces have provided data to the national CDC and were included for analysis in this dispatch.

Specimen Collection

The appropriate clinical specimens (including throat swabs, rectal swabs, fecal samples, vesicular fluid, and/or cerebrospinal fluid) were collected from HFMD patients at the doctors' discretion according to the clinical manifestations and status of each patient. Specimen collection was conducted in a routine way and was not affected by outbreaks.

Specimen Processing

Specimens were placed in sterile viral transport medium. Commercial kits (QIAamp Viral RNA Mini Kit in most cases; QIAGEN, Valencia, CA, USA, and Geneaid Viral RNA Mini Kit, Geneaid Biotech, Taiwan, China) were used to extract RNA per the manufacturer's protocols.

Specimen Testing

Specific primers and probes that targeted pan-enterovirus, EV-A71, CA-V16, and other enteroviruses such as CV-A6 in some cases, were used to test RNA from specimens. All testing results in this dispatch were obtained through reverse transcription PCR (no virus isolation) to ensure consistency and reliability, although results from virus isolation are available for a small number of provinces.

Reference

1. Xing W, Liao Q, Viboud C, Zhang J, Sun J, Wu JT, et al. Hand, foot, and mouth disease in China, 2008–12: an epidemiological study. Lancet Infect Dis. 2014;14:308–18. PubMed http://dx.doi.org/10.1016/S1473-3099(13)70342-6

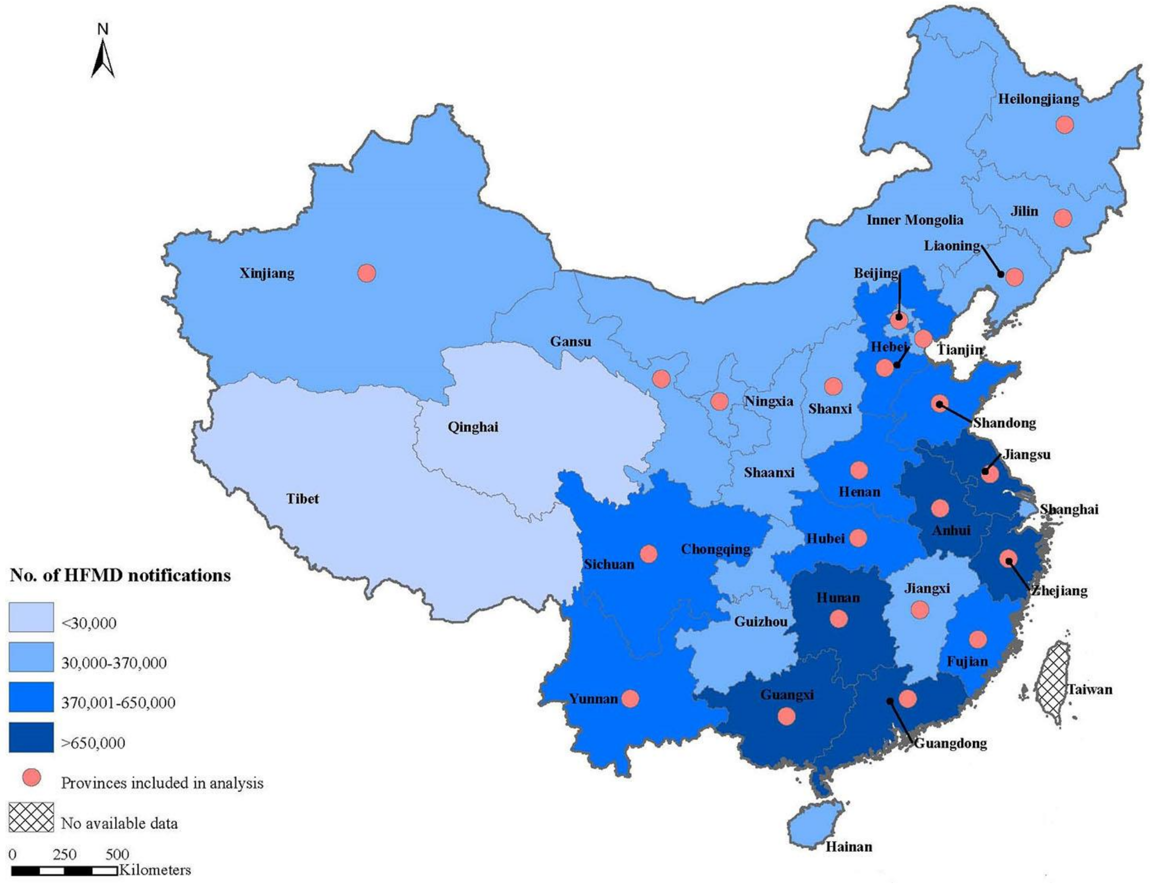

Technical Appendix Figure 1. Provincial distribution of hand, foot and mouth disease notifications, January 2010–December 2016, China, and the 23 provinces included for analysis of laboratory surveillance data.

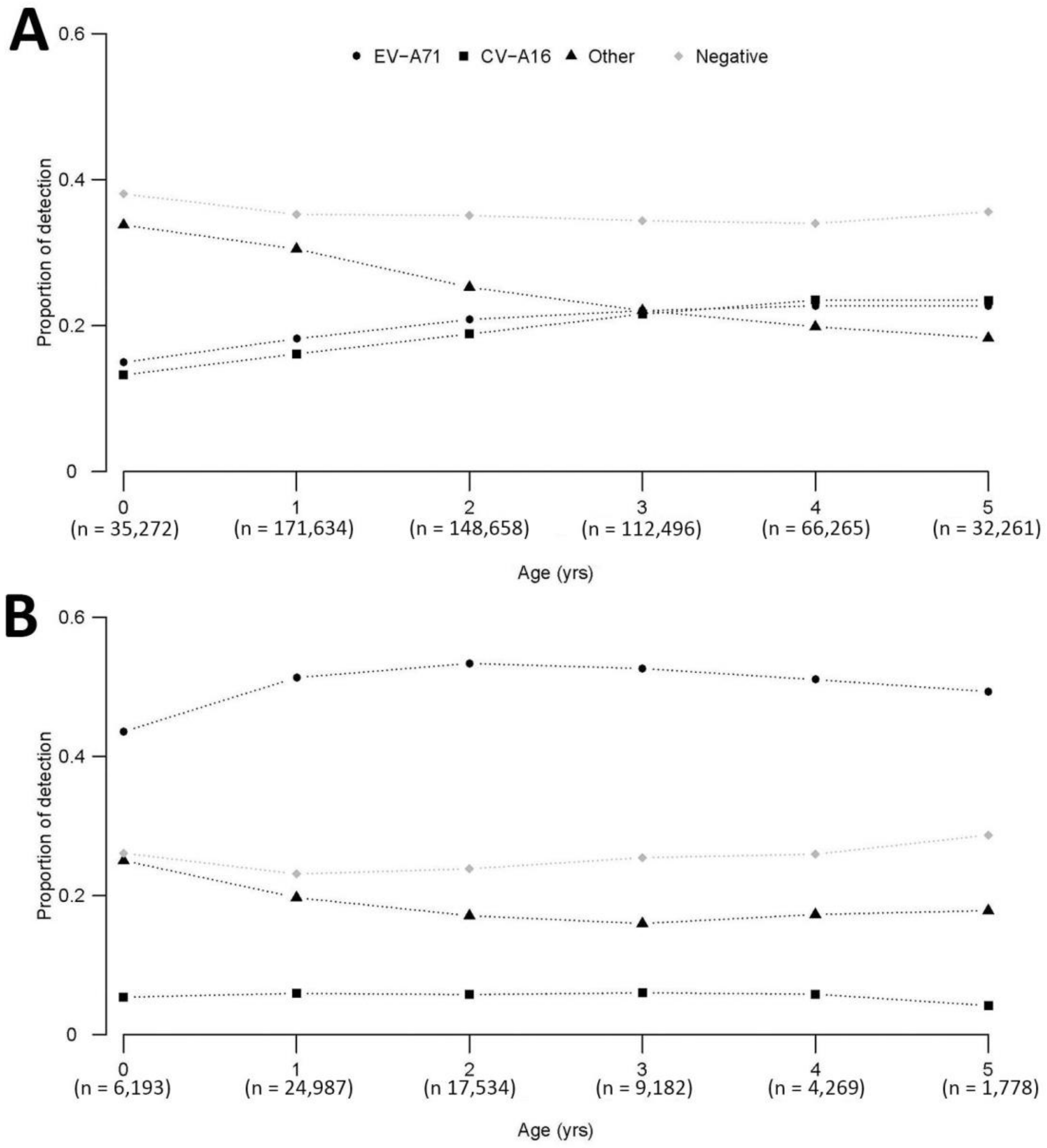

Technical Appendix Figure 2. Yearly proportions of enterovirus detection by serotype among HFMD cases by age, January 2010–December 2016, China: A) mild cases; B) severe cases.

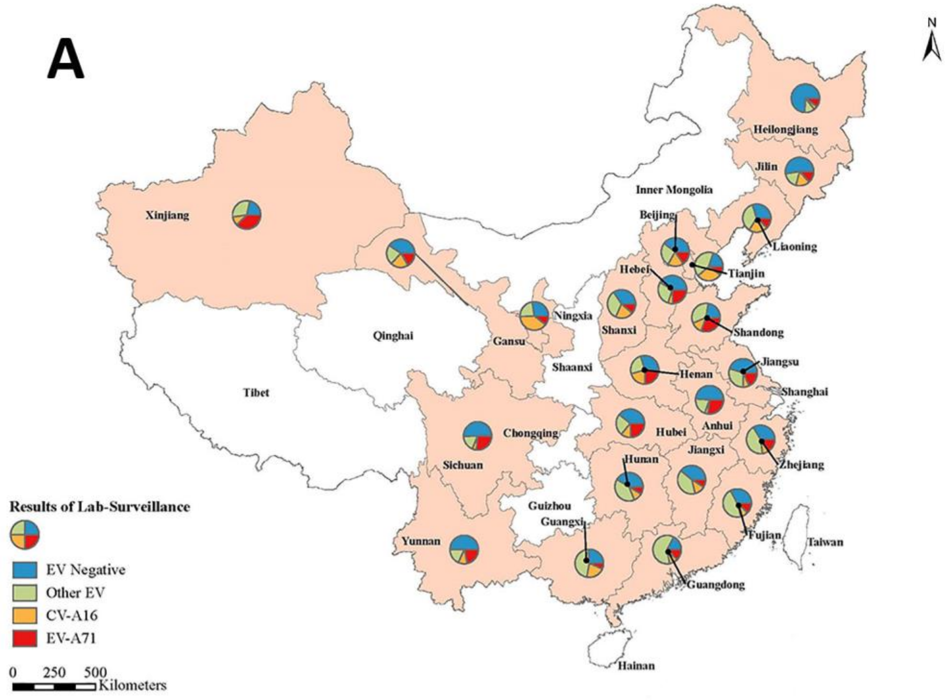

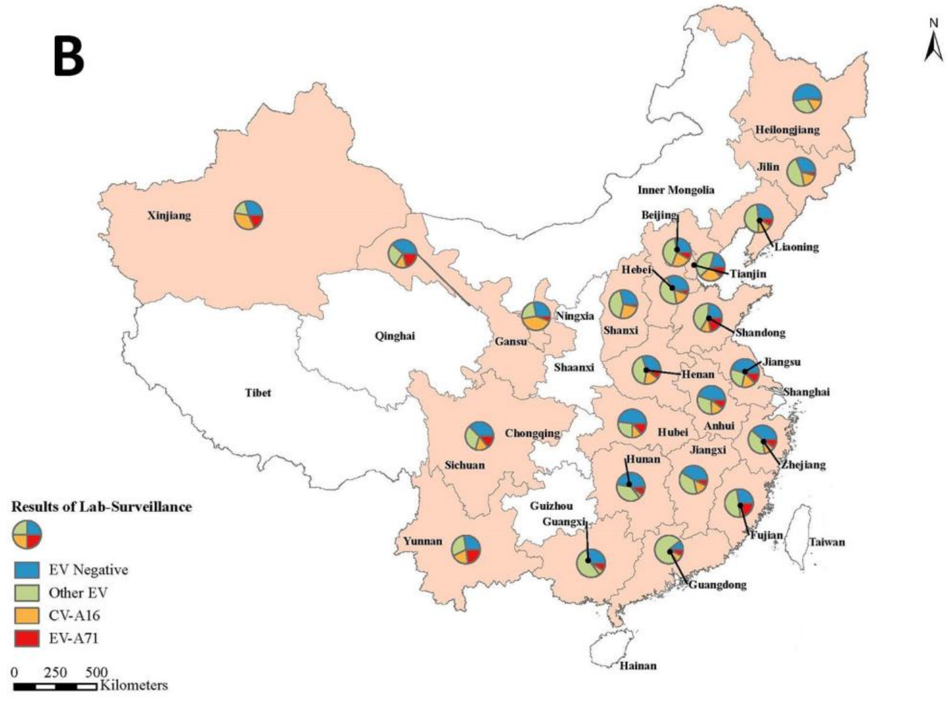

Technical Appendix Figure 3. Detection proportions of EV-A71, CV-A16, and other enteroviruses among mild HFMD cases by province in China: A) 2013; B) 2015.
